# Efficacy of Juzentaihoto for Tumor Immunotherapy in B16 Melanoma Metastasis Model

**DOI:** 10.1155/2017/6054706

**Published:** 2017-02-12

**Authors:** Shintaro Ishikawa, Takako Ishikawa, Chiaki Tezuka, Kazuhito Asano, Masataka Sunagawa, Tadashi Hisamitsu

**Affiliations:** Department of Physiology, School of Medicine, Showa University, Tokyo, Japan

## Abstract

*Introduction*. Medical care for Japanese cancer patients includes Western and Kampo medicines, and treatments with juzentaihoto (JTT) reportedly prevent cancer metastasis and recurrence. In this study, we examined the effects of JTT on natural killer (NK) cell activity and metastasis in combined treatments with anti-PD-1 antibody in a mouse model of melanoma metastasis.* Methods*. C57BL/6 male mice were intravenously injected with B16 melanoma cells (B16 cell) and were given chow containing 3% JTT. In subsequent in vivo experiments, we assessed serum cytokine levels and tumor colony formation in the lungs. Additionally, we assessed NK cell activity in ex vivo experiments.* Results*. JTT significantly suppressed B16 cell metastasis, whereas injection of anti-asialo-GM1 antibody into mice abrogated the inhibitory actions of JTT. JTT significantly increased interleukin- (IL-) 12 and interferon- (IFN-) *γ* levels in serum and induced NK cell activity. It increased the inhibitory actions of the anti-PD-1 antibody on B16 cell metastasis.* Discussion*. These data suggest that JTT inhibits B16 cell metastasis by inducing NK cell activity. Additionally, combination therapy with JTT and anti-PD-1 antibody increased treatment response rates for B16 melanoma.

## 1. Introduction

Melanoma affects 12 people per 100,000 and causes 600 deaths per year in Japan [1]. However, in the United States, approximately 60,000 melanomas are diagnosed and cause 9,000 deaths per year. Moreover, metastatic melanoma is known as an aggressive disease with 5-year survival achieved in only 16% of cases and poor responses to most standard chemotherapies [2]. Patients with regional lymph node involvement have high recurrence rates and numbers of deaths per 100,000 persons remained stable between 1992 and 2011 [3].

Complementary and alternative medicine, including Kampo medicine, compensates for the limitations of Western medicine by stimulating self-defense mechanisms. In Japan, clinicians who have studied both Western and Kampo medicines treat cancer patients with combinations of these medical interventions, and increasing numbers of cancer patients receive outpatient chemotherapy. The traditional medicine juzentaihoto (JTT) has been prescribed widely to retain quality of life in Japan [4]. JTT is a Chinese medicinal preparation comprising 10 herbs and is commonly used as a nutritional agent to improve disturbances and imbalances of homoeostasis. As a Kampo medicine, JTT promotes restoration of physical strength after surgery and alleviates adverse effects of anticancer drugs and radiation therapy. JTT has also been reported to prevent cancer metastasis, occurrence, and recurrence [5–9] and may prolong survival [10, 11].

Targeting of natural killer (NK) cells holds potential in patients with minimal disease, such as initial carcinomatous lesions and hematological cancers [12]. However, the efficacy of NK cells varies between cancers. Previously, we showed that JTT obstructs the metastasis and angiogenesis of B16 melanoma cells [13, 14]. Although the functions of dendritic and NK cells remain unknown, we suggested that uptake of interferon-gamma- (IFN-) *γ* is a common mechanism. Recent studies show that several tumors, including melanoma, have developed the ability to abolish T cell activation and prevent effective T cell antitumor responses. Checkpoint inhibitors, such as anti-programmed cell death-1 (PD-1) antibody, normally promote antitumor immunity by blocking this key negative regulator of T cell activation [15]. Moreover, PD-1 efficacy may be further increased when used in combination with other immune agents.

Herein, we examined the effects of JTT on NK cell activity and metastasis following combined treatment with anti-PD-1 antibody in a B16 transplantation model of metastasis.

## 2. Materials and Methods

### 2.1. Animals

Six-week-old specific pathogen-free C57BL/6 male mice were purchased from Japan CLEA Co. Ltd. (Tokyo, Japan). Animals were maintained in our animal facilities at 25 ± 2°C with 50 ± 2% humidity and a 12 h light/12 h dark cycle. This study was approved by the Showa University Ethics Committee for animal experiments (number 06078).

### 2.2. Reagents

JTT was provided by Tumura Co. Ltd. (Tokyo, Japan) as a pure, preservative-free powder and was thoroughly mixed with a regular powder diet (CE-2) for rats and mice (Japan CLEA Co., Ltd. Tokyo, Japan) at a concentration of 3.0% [13]. To inhibit NK cell activity, anti-asialo-GM1 mouse antibody (014-09801) and normal rabbit IgG (control mouse antibody: 148-09551) were purchased from Wako Pure Chemical Ind. Ltd. (Tokyo, Japan). The anti-asialo-GM1 mouse monoclonal antibody acts against the glycosphingolipid asialo-GM1 antigen, which is expressed on murine NK cells [16]. PD-1 targeting experiments were performed using an anti-PD-1 mouse antibody (RMP1-14) and isotype control rat IgG (control mouse antibody: 2A3), which were purchased from BioXCell (West Lebanon, NH, USA). NK cell viability was assessed using WST-8 reagent (Cell Counting Kit-8; Dojindo Lab., Kumamoto, Japan). NK cells were separated from spleens using Mouse panNK CD49b Selection Kit (Cat. 18755; StemCell Technologies, Vancouver, BC, Canada).

### 2.3. Cell Culture

Cells were cultured in Dulbecco's modified eagle medium (DMEM; Sigma-Aldrich Co., St. Louis, MO, USA) or Roswell Park Memorial Institute 1640 medium (RPMI1640; Sigma-Aldrich Co.) supplemented with 10% heat-inactivated fetal calf serum (FCS; Nihon Bio-Supply Center, Tokyo, Japan) and a penicillin-streptomycin-neomycin (PSN) antibiotic mixture containing penicillin and streptomycin at 5 mg/mL and neomycin at 10 mg/mL (15640; Life Technologies, Inc.). Media were sterilized by passing through 0.2 *μ*m pore filters and were stored at 4°C until use.

B16-F10 mouse melanoma cells (ATCC® CRL-6475™; Rockville, MD, USA) were routinely cultured at 37°C in a humidified atmosphere of 5% CO_2_ and were maintained in DMEM-FCS-PSN. YAC-1 NK-sensitive murine lymphoma cells (ATCC TIB-160™) were cultured in RPMI-1640-FCS-PSN at 37°C in a humidified atmosphere of 5% CO_2_.

### 2.4. Assay of Tumor Cell Metastasis

B16 cells (2 × 10^5^ cells) were injected intravenously into recipient mice in a volume of 50 *μ*L phosphate-buffered saline (PBS). After 21 or 28 days, mice were euthanized under ether anesthesia and numbers of tumor colonies on lung surfaces were counted using a dissecting microscope (SZ-60; OLYMPUS Co. Ltd., Tokyo, Japan).

### 2.5. Influence of NK Cell Depression

Mice were intraperitoneally injected with anti-asialo-GM1 mouse antibody or isotype control mouse antibody (200 mg for both) on every 5th day (on days −1, 4, 9, 14, and 19) from the day before B16 cell inoculation [14]. Tumor colony formation was assessed in lungs on the 21st day after B16 cell injection.

### 2.6. Influence of Combination Therapy with JTT and Anti-PD-1 Antibody

Mice were intraperitoneally injected with anti-PD-1 or isotype control mouse antibodies (200 *μ*g for both) on every 4th day (on days 10, 14, 18, 22, and 26) from the 10th day after B16 cell inoculation [17, 18]. Tumor colony formation in the lung was assessed on the 28th day after B16 cell injections.

### 2.7. Measurement of Cytokines

Interleukin- (IL-) 12 and IFN-*γ* levels in serum and culture supernatants were determined using commercially available enzyme-linked immunosorbent assay (ELISA) kits (M1270; MIF00, R&D Systems, Inc., Minneapolis, MN, USA) according to the manufacturer's recommendations. The sensitivity of the IFN-*γ* assay kit was 2.0 pg/mL and that of the IL-12 assay kit was 2.5 pg/mL. Absorbance at 450 nm was measured using a Multiskan™ GO instrument (Thermo Fisher Scientific Inc. Waltham, MA, USA).

### 2.8. Separation of the NK Cells from Spleen

NK cells were separated using Mouse panNK (CD49b) Selection Kit according to the manufacturer's instructions [19]. Briefly, spleens from recipient animals were homogenized, and cells were resuspended in medium at 1 × 10^8^ cells/mL. Prior to EasySep separations, spleen cells were incubated for 15 min with a positive selection cocktail containing anti-mouse CD49b antibodies. EasySep magnetic nanoparticles were then added to cell-antibody mixtures and were incubated for 10 min at room temperature. PBS containing 2% FCS and 1 mM EDTA was then added to cell suspension to a final volume of 2.5 mL. Samples were then placed into magnetized chambers and were incubated for 5 min. Magnets and tubes were inverted to remove supernatants without disrupting panNK CD49b^+^ cell pellets. After repeating the EasySep procedure three times, tubes were removed from the magnet and the remaining cells were resuspended in culture medium. Positively selected cells were then used in assays to determine NK activity.

### 2.9. Cytotoxicity Assays in NK Cells

NK activities of fresh splenocytes were measured using WST-8 reagent. Briefly, 50 *μ*L aliquots of target YAC-1 cell suspensions (1 × 10^5^ cells/well) were added to 50 *μ*L aliquots of effector cells (fresh nonactivated spleen cells) at various effector : target (E : T) ratios (5 : 1, 20 : 1, and 30 : 1) in 96-well flat-bottomed culture plates. The plates were then centrifuged at 1000 rpm for 5 s and incubated in a 5% CO_2_ incubator at 37°C. After a 4 h incubation, 10 *μ*L aliquots of WST-8 reagent were added, and the plates were incubated for 1 h. Optical density (OD) was determined using a microplate spectrophotometer at 450 nm (reference 655 nm). Cytotoxic activity at each E : T ratio was calculated as follows: cytotoxicity % = [(OD_target_ − (OD_target+effector_ − OD_effector_) − OD_blank_)/(OD_target_ − OD_blank_)] × 100 [20].

### 2.10. Examination of IFN-*γ* Secretion from NK Cells

Separated NK cells were resuspended at a density of 2 × 10^5^ cells/well in DMEM-FCS-PSN and cultured in triplicate in 24-well plates. Subsequently, B16 cells were added to NK cells at a ratio of 1 : 20 (B16/NK), and supernatants were collected after coculture for 24 h [21] and stored at −80°C until use for ELISA measurements of IFN-*γ* concentrations.

### 2.11. Statistical Analysis

Data were expressed as means ± standard deviations (SD). All assays were repeated two times to ensure reproducibility. Differences between control and experimental groups were identified using one-way analysis of variance followed by Scheffe tests and were considered significant when *P* < 0.05.

## 3. Results

### 3.1. Suppression of B16 Cell Metastasis by JTT

**Figure 1:**
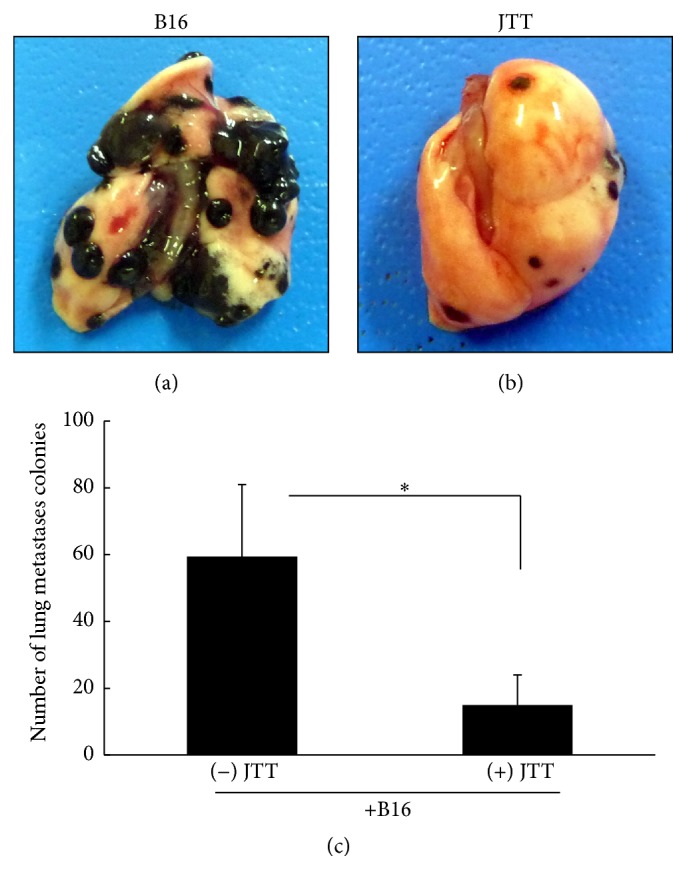
Influence of JTT on B16 melanoma cell metastasis in mice. C57BL/6 mice were orally administered JTT from one week before intravenous injections of 2 × 10^5^ melanoma cells. Mice were sacrificed 3 weeks later, and tumor cell colonies on lung surfaces were counted. (a, b) Image of tumor cell colonies on the lungs; (c) numbers of colonies on the lungs, ^*∗*^*P* < 0.05 versus control, *n* = 10; data are presented as means ± standard deviations (SD).

To examine the influence of JTT on experimental metastasis, we injected 2 × 10^5^ B16 tumor cells intravenously into the caudal vein of mice that were pretreated with 3.0% JTT. Mice were sacrificed 21 days later, and numbers of tumor cell colonies on lung surfaces were counted. As shown in Figure 1, oral administration of 3.0% JTT significantly suppressed B16 cell metastasis.

### 3.2. Influence of NK Cell Inhibition on Tumor Cell Metastasis in JTT-Treated Mice

**Figure 2:**
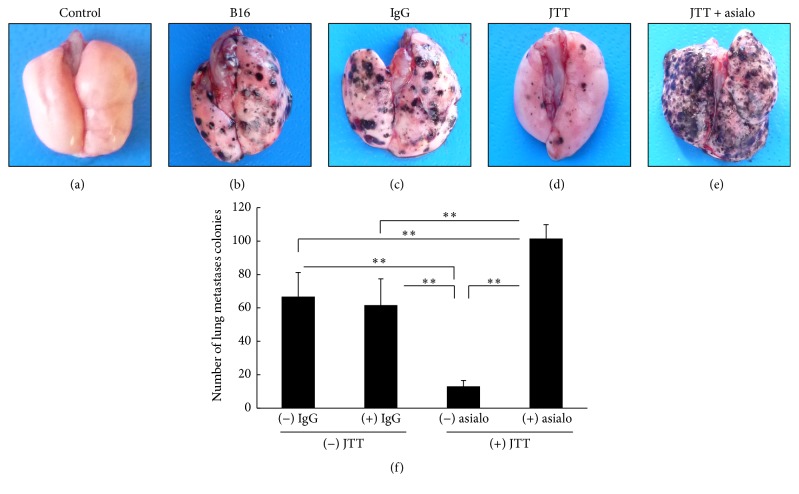
Influence of JTT on IL-12 and IFN-*γ* production in serum. C57BL/6 mice were orally given JTT from one week before intravenous injections of 2 × 10^5^ melanoma cells. Mice were sacrificed 3 weeks later, and serum IL-12 and IFN-*γ* levels were determined. (a) Serum IL-12 contents; (b) serum IFN-*γ* contents; ^*∗*^*P* < 0.05; ^*∗∗*^*P* < 0.01; *n* = 10; data are expressed as means ± SD.

To determine the influence of NK cell inhibition on tumor cell metastasis, mice were treated with anti-asialo-GM1 antibody and intravenous injections of B16 cells. After 21 days, the influence of NK cell depletion by anti-asialo-GM1 antibody was assessed by counting numbers of tumor cell colonies on lung surfaces. As shown in Figure 2, injections of anti-asialo-GM1 antibody into mice abrogated the inhibitory actions of JTT on tumor cell metastases. Additionally, numbers of colonies were significantly higher in mice treated with antibody than in control mice.

### 3.3. NK Cell Activity

**Figure 3:**
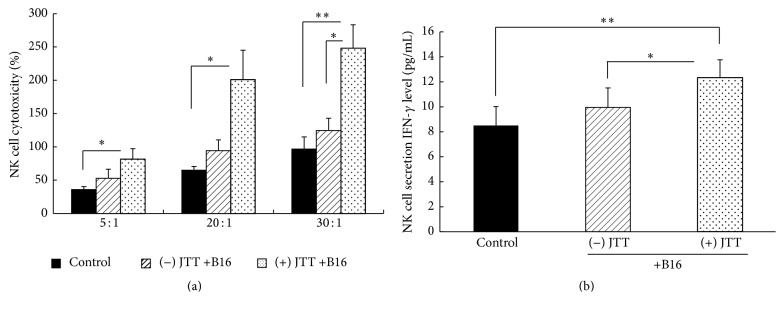
Ex vivo NK cell activity. NK cells were isolated from spleens, and IFN-*γ* secretions and cytotoxic activities were assayed; B16 cell injections, B16 cell injections with JTT treatment, and control mice. (a) IFN-*γ* production in NK cells; isolated NK cells were resuspended at a density of 2 × 10^5^ cells/well in DMEM-FCS-PSN and were cultured in 24-well plates in triplicate. B16 cells were then added to NK cells at a 1 : 20 ratio (B16 : NK). After coculture for 24 h, culture supernatants were collected, and IFN-*γ* levels were measured using enzyme-linked immunosorbent assay (ELISA). (b) Assays of NK cell cytotoxicity toward YCA-1 cells; YAC-1 cells at 1 × 10^5^/well were incubated with fresh spleen cells at fixed E : T ratios for 4 h and NK cell cytotoxicity was measured using a modified WST-8 assay; ^*∗*^*P* < 0.05; ^*∗∗*^*P* < 0.01; *n* = 10; data are expressed as means ± SD.

Initially, we examined cytotoxicity of subject NK cells for target YAC-1 cells. As shown in Figure 3(a), cytotoxicity of NK cells in JTT-treated B16 transplantation mice was significantly greater than in control mice and untreated B16 transplantation mice. Additionally, cytotoxicity for target cells increased in accordance with NK cell numbers. In further experiments, the influence of IFN-*γ* production by NK cells was examined in cocultures with ex vivo B16 cells. Subsequently, ELISA experiments (Figure 3(b)) showed that IFN-*γ* secretion from NK cells was significantly greater in cells from JTT-treated B16 injected mice than in cells from untreated B16 transplantation mice and control mice.

### 3.4. Serum Cytokine Levels

**Figure 4:**
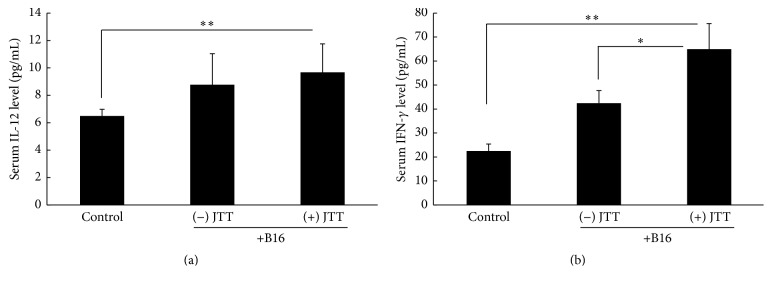
Influence of anti-NK antibody injections on B16 melanoma cell metastasis in mice treated with JTT. C57BL/6 mice were orally given 3.0% JTT for three weeks from one week before injections of 2 × 10^5^ B16 melanoma cells. Anti-asialo-GM1 antibody was injected intraperitoneally and numbers of tumor cell colonies on lungs were counted three weeks later. (a–e) Images of tumor cell colonies on excised lungs; (f) quantification of numbers of colonies on lungs; IgG, normal rabbit IgG; asialo, anti-asialo-GM1 mouse antibody; ^*∗∗*^*P* < 0.01; *n* = 10; data are presented as means ± SD.

Cytokine levels in mouse serum were determined using ELISA kits. As shown in Figure 4, serum IL-12 levels were significantly greater in mice with B16 transplantation tumors than in control mice but were not further increased by JTT administration. However, serum IFN-*γ* levels were significantly greater in mice with B16 transplantation tumors than in control mice and were further elevated by treatments with JTT (Figure 4(b)).

### 3.5. Influence of Combination Treatments with JTT and Anti-PD-1 Antibody on Tumor Metastases

**Figure 5:**
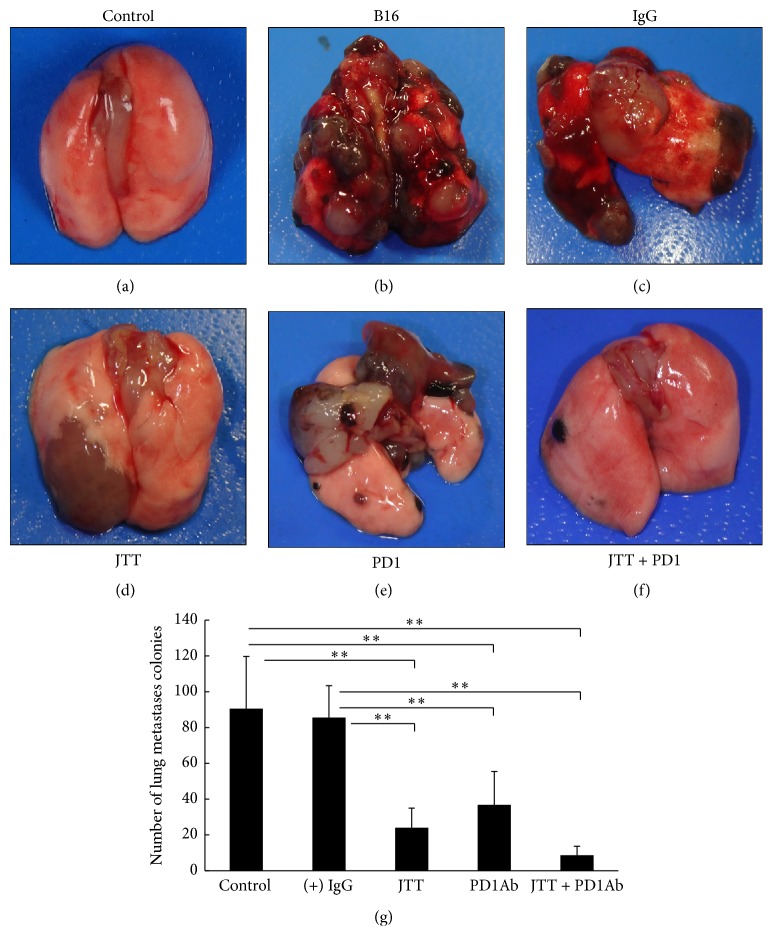
Influence of combined JTT and anti-PD-1 antibody treatments on B16 melanoma cell metastasis in mice. C57BL/6 mice were orally administered with 3.0% JTT for four weeks from the week before 2 × 10^5^ B16 melanoma cells were injected. Anti-PD-1 antibody was injected intraperitoneally and numbers of tumor cell colonies were counted four weeks later. (a–f) Images of tumor cell colonies on lungs; (g) quantification of numbers of colonies on the lungs; IgG, isotype control rat IgG; PD1, anti-PD-1 antibody; ^*∗∗*^*P* < 0.01; *n* = 10; data are presented as means ± SD.

To examine the influence of JTT administration and anti-PD-1 antibody treatments on tumor cell metastasis, B16 transplantation mice were treated with anti-PD-1 antibody, JTT, or the combination, and numbers of tumor colonies on lung surfaces were counted after 28 days. As shown in Figure 5, JTT administration increased the inhibitory actions of anti-PD-1 antibody against tumor cell metastasis in mice.

## 4. Discussion

Herbal medicine is frequently and successfully used as a supplemental therapy for various chronic diseases [22]. Moreover, as a cancer treatment, herbal medicines reportedly prevent the progression of colon carcinoma and gastric and breast cancer and can ameliorate cancer metastases to liver, lung, and bone tissues [23]. However, the mechanisms by which herbal medicines improve clinical conditions for cancer patients, including cancer metastasis, remain poorly understood. Herein, we examined anticancer mechanisms of the herbal medicine JTT in mice with 21-day-old B16 cell transplantation tumors.

The current results show that oral administration of JTT inhibits B16 cell colony formation on the lung surface following intravenous injections of tumor cells (Figure 1). The mechanisms that lead to arrest of tumor cell growth and metastasis are diverse and reflect tumor cell death, apoptosis, and immune-mediated cancer regression. Previous studies indicate little chemotherapeutic cytotoxicity of JTT toward B16 cells [14]. However, because NK cell activity contributes to inhibition of B16 cell metastasis, we examined the influences of JTT on NK cell survival and activity. In our initial experiments, we investigated the effects of JTT on B16 cell lung metastases in the presence of the anti-asialo-GM1 antibody, which depletes NK cell numbers in mice. As indicated in Figure 2, JTT administration in NK cell-depleted animals did not inhibit B16 cell metastases. However, upon further examination of the influence of JTT on NK cell cytotoxicity, NK cell after JTT treatments significantly increased the cytotoxicity for YAC-1 cell (Figure 3(a)). Additionally, ex vivo NK cells from JTT-treated B16 transplantation mice had increased IFN-*γ* secretion when cocultured with B16 cells (Figure 3(b)). NK cells are potent effectors that, unlike T lymphocytes, eliminate target cells spontaneously without prior sensitization. Moreover, NK cells have strong cytolytic activity against virus-infected and tumor cells. Specifically, activated NK cells were shown to exert immunomodulatory effects by producing cytokines such as IFN-*γ* and tumor necrosis factor and also directly kill target cells by releasing perforins and granzymes [24]. Taken together, these studies suggest that JTT inhibits B16 cell metastasis by inducing NK cell activity. NK cells shape the immune response by interacting with dendritic cells (DC), and crosstalk between DC and NK cells is mediated by secretions of several cytokines, including IL-12 and IFN-*γ* [25]. Therefore, we examined the roles of these key cytokines in vivo and showed increased serum levels following JTT treatments in B16 cell transplantation mice. NK cells are key mediators in innate responses against tumors, with cytotoxic activity and production of substantial quantities of IFN-*γ* and other proinflammatory cytokines. Moreover, crosstalk between DC and NK cells involves NKp30 and DNAM-1 and subsequent secretion of cytokines such as IL-12 and IFN-*γ* [5]. Additionally, NK cells positively and negatively influence anticancer responses by regulating immune crosstalk with DC and T cells [24].

In 1992, Ishida et al. showed that the protein PD-1 induces T cell death [26]. In more recent studies, anti-PD-1 antibodies were used for their strong antitumor effects against several malignancies, including melanoma, and studies of combined effects with various immunotherapies have been conducted widely [27, 28]. Therefore, we examined the effects of combined treatments with JTT and an anti-PD-1 antibody on B16 transplantation mice. These experiments showed that administration of JTT or anti-PD-1 antibody inhibited the formation of B16 metastases on lung surfaces (Figure 5). Although the combination did not result in significant additive effects, there seems to be no possibility that JTT offsets the action of anti-PD-1 antibodies at least. Because JTT likely has no direct effects on the actions of anti-PD-1 antibodies, it may prevent tumor-mediated inactivation of immune cells such as NK cells or cytotoxic T lymphocytes.

Several cancers, including melanoma, have been shown to inactivate T cells and prevent the ensuing antitumor responses, and inhibitors such as anti-PD-1 antibodies can reverse this immune suppression and restore T cell activation. Accordingly, the present anti-PD-1 antibody has been shown to promote antitumor immunity. NK cells participate in early immune responses against melanoma and also contribute to adaptive immune responses through cytokine secretions and crosstalk with DC [29]. Moreover, IL-18 secretion by tumor cells upregulates PD-1 expression on NK cells and PD-L1 expression on DC and may reduce numbers and actions of mature NK cells [30, 31]. Previous studies show that JTT induces NK cell activity and IFN-*γ* secretion in the presence of the anti-PD-1 antibody nivolumimab, suggesting that JTT helps to circumvent PD-1 mediated inhibition of NK cells. In a clinical trial of combined JTT and anti-PD-1 antibody treatments, a subset of patients responded to single-agent blockade, while the combined treatment showed the potential to improve response rates. Hence, although combination therapies may also lead to immune related adverse events, its therapies appear to be a means of increasing response rates for cancer treatment.

Previous data suggest that IL-12 from immunocytes such as DC is an important mediator of JTT-induced NK activity. Because various cytokines, such as IL-2, IL-12, IL-15, IL-18, IL-21, and type I IFNs, affect the mobility and cytotoxicity of NK cells [32], future examinations are necessary to determine trends of these cytokines under the present conditions. Furthermore, bidirectional interactions between NK and tumor cells remain poorly characterized.

Lajoie et al. [33] recently showed that NKp46-activating antibodies activate NK cells and lead to degranulation and cytokine secretion, as indicated by CD107a and CD16 expression, and IFN-*γ* secretion, respectively. Melanoma cells contrive resistance to NK cell-mediated death by increasing the expression of HLA class I molecules [34, 35] and decreasing the expression of MICA and NCR, which are ligands that induce cytotoxic activities of NK cells [36–38]. Additionally, downregulation of DNAM-1-, NKp46-, NKp30-, and NKG2D-activating receptors on NK cells reportedly contributes to the escape of melanoma cells [39–41]. However, in our current study, IFN-*γ* secretion was increased following JTT treatments of the ex vivo NK cells from B16 transplantation mice (Figure 3(b)). Hence, JTT may not affect antitumor receptors on NK cells in the presence of melanoma, suggesting that JTT circumvents melanoma mediated downregulation of activating receptors on NK cells. However, future examinations are necessary to clarify the effects of JTT on these molecular changes.

## 5. Conclusion

These present data suggest that JTT inhibits B16 cell metastasis by inducing NK cell activity. Additionally, combination therapy with JTT and anti-PD-1 antibody may increase treatment response rates for B16 melanoma.
